# Adolescent endogenous sex hormones and breast density in early adulthood

**DOI:** 10.1186/s13058-015-0581-4

**Published:** 2015-06-04

**Authors:** Seungyoun Jung, Brian L. Egleston, D. Walt Chandler, Linda Van Horn, Nola M. Hylton, Catherine C. Klifa, Norman L. Lasser, Erin S. LeBlanc, Kenneth Paris, John A. Shepherd, Linda G. Snetselaar, Frank Z. Stanczyk, Victor J. Stevens, Joanne F. Dorgan

**Affiliations:** Department of Epidemiology and Public Health, University of Maryland School of Medicine, Howard Hall 102E, Baltimore, MD 21201 USA; Fox Chase Cancer Center, 333 Cottman Avenue, Philadelphia, PA 19111 USA; Esoterix Inc, 4301 Lost Hills Road, Calabasas Hills, CA 91301 USA; Department of Preventive Medicine, Feinberg School of Medicine, Northwestern University, 303 E Chicago Avenue, Chicago, IL 60611 USA; Department of Radiology, University of California, San Francisco, 500 Parnassus Avenue, San Francisco, CA 94143 USA; Dangeard Group, 580 W Remington Drive, San Francisco, CA 94087 USA; Department of Medicine, Rutgers New Jersey Medical School, 185 S Orange Avenue, Newark, NJ 07103 USA; Kaiser Permanente Center for Health Research, 3800 N Interstate Avenue, Portland, OR 97227 USA; Department of Pediatrics, Louisiana State University School of Medicine, 1901 Perdido Street, New Orleans, LA 70112 USA; Department of Epidemiology, University of Iowa, 200 Hawkins Drive, Iowa City, IA 52242 USA; Department of Obstetrics and Gynecology, University of Southern California Keck School of Medicine, 1975 Zonal Avenue, Los Angeles, CA 90033 USA

## Abstract

**Introduction:**

During adolescence the breasts undergo rapid growth and development under the influence of sex hormones. Although the hormonal etiology of breast cancer is hypothesized, it remains unknown whether adolescent sex hormones are associated with adult breast density, which is a strong risk factor for breast cancer.

**Methods:**

Percentage of dense breast volume (%DBV) was measured in 2006 by magnetic resonance imaging in 177 women aged 25–29 years who had participated in the Dietary Intervention Study in Children from 1988 to 1997. They had sex hormones and sex hormone-binding globulin (SHBG) measured in serum collected on one to five occasions between 8 and 17 years of age. Multivariable linear mixed-effect regression models were used to evaluate the associations of adolescent sex hormones and SHBG with %DBV.

**Results:**

Dehydroepiandrosterone sulfate (DHEAS) and SHBG measured in premenarche serum samples were significantly positively associated with %DBV (all *P*_trend_ ≤0.03) but not when measured in postmenarche samples (all *P*_trend_ ≥0.42). The multivariable geometric mean of %DBV across quartiles of premenarcheal DHEAS and SHBG increased from 16.7 to 22.1 % and from 14.1 to 24.3 %, respectively. Estrogens, progesterone, androstenedione, and testosterone in pre- or postmenarche serum samples were not associated with %DBV (all *P*_trend_ ≥0.16).

**Conclusions:**

Our results suggest that higher premenarcheal DHEAS and SHBG levels are associated with higher %DBV in young women. Whether this association translates into an increased risk of breast cancer later in life is currently unknown.

**Clinical trials registration:**

ClinicalTrials.gov Identifier, NCT00458588 April 9, 2007; NCT00000459 October 27, 1999

## Introduction

Breast density is a measure of the relative proportion of glandular and stromal tissue to fatty tissue in the breasts [1, 2] and is a strong risk factor for breast cancer [2]. Women with high breast density have a four- to sixfold increased risk of breast cancer [2]. Although reproductive and menstrual factors [3] and exogenous hormone use [4, 5] are positively associated with breast density, associations of endogenous sex hormones with breast density are inconsistent. Three [6–8] out of seven studies in premenopausal women [6–12] found no association between any of the sex hormones measured and breast density, whereas others reported significant associations with estrogens [8, 10, 12], progesterone [9], and sex hormone-binding globulin (SHBG) [10, 11]. Results for postmenopausal women [8, 13–23] are similarly conflicting and largely null.

Previous studies mainly enrolled middle-aged and older women. It remains unknown whether adolescent sex hormone levels are associated with breast density and breast cancer risk during adulthood. Adolescence is a time of rapid breast growth and development and increased susceptibility of breast tissue to carcinogens [24–26]. Modulating estrogen levels during puberty was reported to alter mammary ductal morphology in animal studies [27, 28]. Hormonal contraceptive use beginning at 12–17 years of age was associated with higher dense breast volume at 25–29 years of age than hormonal contraceptive use beginning at 22–28 years [29]. In a pooled analysis, a longer reproductive period that was due to a younger age at menarche was associated with a larger increase in breast cancer risk than a longer reproductive period that was due to older age at menopause [30]. Collectively, the hormonal milieu during adolescence may be important in determining breast morphology and breast cancer risk.

To our knowledge, no prior study has evaluated associations between adolescent sex hormones and adult breast density, possibly because of few cohorts initiated at a young age. Therefore, we prospectively evaluated associations of adolescent serum sex hormones with breast density measured in young women in the Dietary Intervention Study in Children (DISC) and the DISC 2006 (DISC06) Follow-up Study [29, 31, 32].

## Methods

### Study design and population

DISC was a two-armed, multicenter, randomized, controlled clinical trial evaluating the efficacy and safety of a dietary intervention to reduce serum low-density lipoprotein cholesterol (LDL-C) in children [33, 34]. Between 1988 and 1990, 663 prepubertal children aged 7–10 years with elevated serum LDL-C were recruited at six clinical centers, and were randomized into two groups: a behavioral diet intervention group to reduce fat intake and a usual-care control group. In 1990, a hormone study ancillary to the DISC was initiated to assess the effect of the diet intervention on serum sex hormone levels [32, 35]. The intervention continued until 1997, when participants’ mean age was 16.7 years [36]. From 2006 to 2008, the DISC06 Follow-up Study was conducted to assess the long-term effect of the diet intervention during childhood and adolescence on biomarkers associated with breast cancer risk [31].

Of the 301 female DISC participants, 286 had sex hormones measured at baseline or follow-up visits that took place at year 1, 3, 5 and last visits (approximately 7) years after randomization; none were pregnant or had used oral contraceptives within 3 months before blood collection [32]. Among these, 257 participated in the DISC06 Follow-up Study. We excluded women who were pregnant or breastfeeding at or within 12 weeks before the DISC06 follow-up visit, had breast augmentation or reduction surgery, or who had a technically unacceptable or missing magnetic resonance imaging (MRI) breast scan. Our final sample included a total of 177 women with breast density information and at least one measurement of adolescent serum sex hormones during the DISC trial; because of few measurements of sex hormones at baseline (N = 38), baseline hormone data were only used for participants missing hormone data at the year 1 DISC visit.

For the DISC trial, assent from DISC participants and informed consent from parents and guardians were obtained. All participants in the DISC06 Follow-up Study provided informed consent. The institutional review boards at participating centers approved all DISC protocols (see the list of the institutional review boards in the acknowledgements).

### Data collection

At all clinic visits during the DISC trial and a single clinic visit in the DISC06 Follow-up Study, participants completed questionnaires inquiring demographic and lifestyle information, medical and reproductive history, and medication use. Weight and height were measured using a standardized protocol [37]; body mass index (BMI) was calculated as weight (kg)/height (m^2^). Diet was assessed by three nonconsecutive 24-hour dietary recalls over a 2-week period. Sexual maturation was assessed by Tanner stage [38] and onset of menses was ascertained annually during the DISC trial. We determined the days until the start of the next menses that corresponded to the date of blood collection from menstrual calendars completed by postmenarcheal participants for 6 weeks after their blood collections.

#### Blood sampling

At baseline and at year 1, 3, 5 and last follow-up visits in the DISC trial [32], a single blood sample was collected in the morning from participants who had fasted overnight. After standing at room temperature for 45 minutes to allow complete clotting, serum was separated by centrifugation, aliquoted into glass vials, and stored at −70 °C.

#### Laboratory assays

All laboratory assays were performed at Esoterix, Inc. (Calabasas Hills, CA, USA) using standard procedures. Steroid sex hormones were measured by radioimmunoassays (RIAs) following extraction (androstenedione, progesterone) and chromatography (estradiol, estrone, testosterone) or directly (dehydroepiandrosterone sulfate (DHEAS)) [39] Estrone sulfate and DHEAS were measured following enzymolysis as estrone and DHEA, respectively. SHBG was measured by an immunoradiometric assay [40]. Non-SHBG-bound estradiol concentration was calculated by multiplying the total estradiol concentration by the percentage of non-SHBG-bound estradiol measured by ammonium sulfate precipitation [41].

Analytical samples collected from the same clinic visit were assayed together in the same assay batches. Masked quality control (QC) samples that were aliquots from three serum QC pools were included in each batch [39]. The within-visit coefficients of variation (CV), estimated from QC samples, were 8–29 % for estradiol, 12–31 % for estrone, 12–17 % for estrone sulfate, 4–10 % for progesterone, 8–17 % for androstenedione, 5–9 % for DHEAS, 9–22 % for testosterone, and 15 % for SHBG. Low concentrations may have contributed to higher CVs for some hormones.

#### Breast density measurement

Breast density was measured using noncontrast MRI on a whole-body 1.5 Tesla or higher field-strength MRI scanner with a dedicated breast-imaging radiofrequency coil [37]. At all clinics, MRI technologists followed a common image acquisition protocol and were trained to recognize and correct failures caused by incomplete fat suppression, motion artifacts, and inadequate breast coverage. Sites were certified for MRI images following acceptable image quality on three volunteers. Dr. C. Klifa processed all MRI images using customized software to identify the chest wall-breast tissue boundary and skin surface and to separate breast fibroglandular and fatty tissue [42]. For images with incomplete or failed fat saturation, manual delineation was performed.

The total breast volume and absolute dense breast volume (ADBV), which quantifies fibroglandular tissue volume, were measured. The absolute nondense breast volume (ANDBV) was calculated by subtracting ADBV from total breast volume. The percentage of dense breast volume (%DBV) was calculated as the ratio of ADBV to total breast volume. The density measures of both breasts were averaged for analysis. We considered %DBV as primary outcome given the established association of mammographic %DBV with breast cancer [43].

### Statistical analysis

Breast density measures were initially described using nonparametric statistics, and prior to modeling were log-transformed to improve normality. Because hormone levels change during adolescence [44, 45], we standardized hormone data to have a comparable distribution across the DISC clinic visits. We first categorized hormone values into deciles based on the distribution of the hormone within each visit. Separately for visits that occurred before and after menarche, the visit-specific deciles of hormone concentrations were then regressed on predictors of adolescent hormone levels including age, BMI z-score, and visit number using a linear regression estimated by generalized estimating equations with an exchangeable correlation structure; after menarche, days until the start of the next menses was also included as a cubic spline. Residuals from these models were then added to the mean of the visit-specific decile to give standardized visit-specific deciles. These standardized visit-specific deciles were averaged for each participant separately for visits before and after menarche for the analysis of androgens and SHBG. For estrogens and progesterone postmenarche visits were further averaged separately for the follicular (0 or >14 days until start of next menses) and luteal (1–14 days until start of next menses) phase of the menstrual cycle. Higher quartiles of standardized sex hormone or SHBG values represent higher levels throughout the pre- or postmenarcheal periods.

We calculated the geometric mean of %DBV, ADBV, and ANDBV and 95 % confidence interval (CI) across quartiles of standardized hormone concentrations by exponentiating the coefficients from a multivariable linear mixed-effects regression model with robust standard errors [46]. The clinic was included as a random effect. The following potential confounders [29, 37, 47] were included as fixed effects: race, education level, treatment group, BMI at DISC06 follow-up, duration of hormonal contraceptive use, and parity (see Table 2 for categorization of covariates). Test for trend was from a Wald test using quartile medians. Tests for interaction by treatment assignment were conducted by including cross-product terms in the fully adjusted models.

In sensitivity analyses, we conducted analyses stratified by DISC visit (baseline, ≤year 1, year 3, 5, and the last trial visit), stages of breast development (≤3 and ≥4), and treatment assignment (intervention and usual care). We also excluded participants with more than 33 days from blood draw to their next menses during the DISC trial and those using hormonal contraceptives at DISC06 follow-up visits.

Analyses were conducted using SAS (Cary, NC, USA) and STATA (College Station, TX, USA) statistical software. All tests were two sided. *P* values <0.05 were considered statistically significant.

## Results

Among 177 women available for analyses, the mean age was 27.1 years at the DISC06 follow-up visit (Table 1). The majority were white (91.5 %), nulliparous (71.2 %), and ever users of hormonal contraceptives (93.8 %). The mean BMI was 25.4 kg/m^2^. The mean age at menarche was 12.8 years. The median and interquartile range of %DBV was 24.3 (9.7–41.2). The mean ages at blood collection were 10.2, 12.1, 14.2, and 16.6 years at ≤year 1, year 3, year 5, and the last DISC trial visits, respectively. Additional information on lifestyle, diet, pubertal characteristics, and levels of sex hormones and SHBG during DISC trial visits is summarized in Table S1 in Additional file 1. All girls were prepubertal at baseline. Tanner stages of sexual maturation and the percentage of girls who were postmenarcheal gradually increased over the course of the trial; median levels of sex hormones varied accordingly. In contrast, BMI z-scores were similar across visits.

**Table 1:** Participant characteristics (*N* = 177)

Characteristics	*N*	Mean (SD)
Age at blood collection during adolescence (yrs)		
at ≤1 year visit	115	10.2 (0.6)
at year 3 visit	167	12.1 (0.60)
at year 5 visit	143	14.2 (0.63)
at last visit^a^	143	16.6 (0.88)
Age at breast MRI (yrs)	177	27.1 (1.01)
BMI at breast MRI (kg/m^2^)	177	25.4 (5.38)
Duration of hormonal contraceptive use (yrs)	177	5.2 (3.68)
Age at menarche (yrs)	177	12.9 (1.26)
	*N*	Percentage
*Race*		
White	162	91.5 %
Nonwhite	15	8.5 %
*Education*		
Bachelor's degree	93	52.5 %
Graduate degree	24	33.9 %
Other	60	13.6 %
*Number of pregnancies*		
0	126	71.2 %
≥1	51	28.8 %
*Family history of breast cancer*		
No	167	96.5 %
Yes	6	3.5 %
*Hormonal contraceptive use*		
Never	11	6.2 %
Former	65	36.7 %
Current	101	57.1 %
Breast density measures	*N*	Median (IQR)
Percent dense breast volume (%)	177	24.3 (9.7-41.2)
Absolute dense breast volume (cm^3^)	177	50.0 (93.3-140.3)
Absolute nondense breast volume (cm^3^)	177	299.2 (157.8-484.9)

*Abbreviations: SD* standard deviation, *MRI* magnetic resonance imaging, *BMI* body mass index, *IQR* interquartile range

^a^Last visit for blood collection occurred a median of 7 years after randomization in DISC

Tables 2 and 3 show associations of adult %DBV with sex hormones and SHBG during adolescence adjusted for adult BMI and other potential confounding factors. Results from the multivariable model without adult BMI were generally similar, possibly due to its high correlation with childhood BMI that is adjusted for when standardizing hormone data. DHEAS and SHBG concentrations before menarche were significantly positively associated with %DBV. The multivariable adjusted geometric mean of %DBV was 16.7 % in women in the lowest quartile of premenarcheal DHEAS and 19.6 %, 16.6 %, and 22.1 % in women in the second, third, and highest quartile of premenarcheal DHEAS, respectively (*P*_trend_ <0.001). The multivariable geometric means of %DBV in women increasing quartiles of premenarcheal SHBG quartiles were 14.4 % to 18.7 %, 18.3 % and 24.3 % (*P*_trend_ = 0.03). In contrast, adolescent DHEAS and SHBG concentrations after menarche were not associated with adult %DBV. Estrogen, progesterone, androstenedione, and testosterone concentrations in serum collected during adolescence before or after menarche were not associated with adult %DBV. Results generally were similar when nonstandardized hormone levels measured separately at each clinic visit were examined in association with %DBV (data not shown). An exception was follicular phase total and non-SHBG estradiol measured at the last DISC trial visit when women in the highest quartiles of these hormones had significantly lower %DBV compared to women in the lowest quartiles. These associations were not consistently observed for postmenarcheal samples across clinic visits and could be due to chance.

**Table 2:** Multivariate^a^-adjusted geometric mean and 95 % confidence interval (CI) for percent dense breast volume by quartiles of estrogens and progesterone at pre-^b^ and postmenarcheal^c^ period

	Premenarche		Postmenarche			
Quartiles			Follicular phase^d^		Luteal phase^d^	
	Mean	(95 % CI)	Mean	(95 % CI)	Mean	(95 % CI)
Estradiol		N = 153		N = 110		N = 88
Q1	17.3	(11.1-21.0)	17.1	(11.7-25.0)	16.7	(11.6-23.9)
Q2	18.3	(12.4-27.0)	23.6	(19.8-28.1)	15.3	(10.7-21.9)
Q3	17.6	(14.6-21.2)	15.0	(10.3-21.8)	19.4	(15.2-24.8)
Q4	21.5	(18.9-24.4)	17.5	(13.8-22.2)	17.8	(12.4-25.6)
*P-*trend^e^	0.26		0.42		0.43	
Non-SHBG-bound estradiol		N = 151		N = 106		N = 88
Q1	17.8	(12.1-26.2)	17.6	(13.5-23.0)	18.2	(12.7-26.2)
Q2	20.3	(14.0-29.6)	22.7	(16.1-32.2)	12.3	(10.5-14.3)
Q3	16.3	(11.7-22.9)	14.9	(12.6-17.7)	22.0	(17.8-27.2)
Q4	19.6	(16.6-23.1)	17.7	(13.2-23.8)	18.0	(13.1-24.8)
*P*-trend^e^	0.78		0.24		0.43	
Estrone		N = 153		N = 110		N = 88
Q1	18.1	(12.3-26.6)	15.5	(12.1-19.8)	16.2	(13.5-20.9)
Q2	18.0	(14.0-23.2)	19.7	(13.1-29.7)	17.2	(10.1-29.4)
Q3	21.4	(15.3-29.8)	18.3	(14.5-23.0)	17.4	(11.9-25.4)
Q4	17.1	(13.0-22.5)	18.8	(14.3-24.8)	17.6	(12.6-24.5)
*P*-trend^e^	0.93		0.43		0.71	
Estrone sulfate		N = 146		N = 110		N = 88
Q1	21.2	(14.8-30.4)	19.3	(12.3-30.1)	19.4	(13.9-27.0)
Q2	15.2	(9.9-23.5)	14.9	(12.1-18.2)	13.8	(12.2-15.8)
Q3	15.7	(11.1-22.2)	15.7	(12.2-20.3)	18.7	(14.3-24.4)
Q4	22.5	(18.9-26.9)	23.4	(21.2-25.8)	17.7	(13.1-23.8)
*P*-trend^e^	0.79		0.49		0.73	
Progesterone				N = 110		N = 84
Q1	–		17.0	(11.0-24.8)	16.4	(14.4-18.6)
Q2	–		21.0	(17.0-26.2)	16.7	(13.1-21.3)
Q3	–		16.5	(12.9-24.4)	19.5	(11.5-33.1)
Q4	–		15.8	(13.6-18.8)	17.6	(11.2-27.8)
*P*-trend^e^			0.81		0.38	

*Abbreviations: CI* confidence interval, *SHBG* sex hormone-binding globulin

^a^Geometric means and 95 % CI are estimated from linear mixed-effects models including clinic as a random effect and adjusted for treatment group (diet intervention group, usual-care control group), BMI (kg/m^2^, continuous), parity (0 and >0), duration of hormone use (yrs, continuous), race (white and nonwhite), and education (bachelor's degree, graduate school and other) as fixed effects

^b^The premenarcheal estrogen levels were standardized by adjusting for age at visit, BMI z-score at visit, and visit number in years after randomization by residual method

^c^The postmenarcheal estrogens and progesterone levels were standardized by adjusting for age at visit, BMI z-score at visit, visit number in years after randomization and days from blood draw to start of next menses as a cubic spline by residual method

^d.^Days 1 through 14 before next menses were defined as luteal, whereas days 0 and greater than 14 were defined as follicular

^e^ *P*-test for trend was conducted by modeling the quartile medians of standardized hormone concentrations as a continuous term in linear mixed-effects models and calculating the Wald test statistic

**Table 3:** Multivariate^a^-adjusted geometric mean and 95% confidence interval (CI) for percent dense breast volume by quartiles of androgens and SHBG at pre-^b^ and postmenarcheal^b^ periods

Quartiles	Premenarche		Postmenarche		All periods	
	Mean	(95 % CI)	Mean	(95 % CI)	Mean	(95 % CI)
Androstenedione		N = 153		N = 154		N = 174
Q1	20.2	(17.2-23.7)	17.9	(14.6-22.1)	19.2	(16.5-22.3)
Q2	18.8	(14.9-23.8)	19.4	(14.8-25.5)	19.2	(14.7-24.9)
Q3	18.1	(13.5-24.1)	18.0	(14.4-22.3)	18.0	(15.2-21.3)
Q4	17.4	(11.0-27.6)	17.9	(13.7-23.5)	18.6	(14.1-24.6)
*P*-trend^c^	0.30		0.88		0.69	
DHEAS		N = 153		N = 156		N = 176
Q1	16.7	(12.7-22.0)	19.9	(13.6-29.1)	18.7	(14.2-24.5)
Q2	19.6	(14.2-26.9)	16.3	(12.1-22.1)	17.5	(14.6-20.9)
Q3	16.6	(13.2-20.9)	17.0	(14.5-20.0)	17.5	(15.1-20.3)
Q4	22.1	(17.3-28.0)	20.3	(17.9-22.9)	21.5	(18.2-15.4)
*P*-trend^c^	<0.001		0.84		0.10	
Testosterone		N = 150		N = 156		N = 175
Q1	17.6	(14.8-20.9)	16.7	(14.1-19.7)	18.5	(15.2-22.6)
Q2	20.5	(18.4-22.7)	18.6	(14.8-23.3)	19.2	(16.3-22.7)
Q3	18.0	(12.0-26.9)	18.2	(12.5-26.4)	18.7	(14.7-23.8)
Q4	17.7	(11.5-27.2)	19.8	(17.4-22.6)	18.2	(14.1-23.6)
*P*-trend^c^	0.90		0.16		0.87	
SHBG		N = 153		N = 156		N = 176
Q1	14.4	(10.2-20.4)	15.7	(11.0-22.6)	15.5	(12.8-18.7)
Q2	18.7	(15.3-22.8)	17.1	(12.9-22.7)	17.5	(13.3-23.1)
Q3	18.3	(14.6-22.9)	25.0	(18.0-24.6)	22.1	(20.2-24.2)
Q4	24.3	(19.6-30.1)	16.7	(14.8-18.8)	20.5	(15.6-26.9)
*P*-trend^c^	0.03		0.42		0.05	

*Abbreviations: CI* confidence interval, *DHEAS*, dehydroepiandrosterone sulfate; *SHBG*, sex hormone-binding globulin

^a^Geometric means and 95 % CI are estimated from linear mixed-effects models including clinic as a random effect and adjusted for treatment group (diet intervention group, usual-care control group, BMI (kg/m^2^, continuous), parity (0 and >0), duration of hormone use (yrs, continuous), race (white and nonwhite), and education (bachelor's degree, graduate school and other) as fixed effects

^b^The androgens and SHBG levels were standardized by adjusting for age at visit, BMI z-score at visit, and visit number in years after randomization by residual method

^c^ *P*-test for trend was conducted by modeling the quartile medians of standardized hormone concentrations as a continuous term in linear mixed-effects models and calculating the Wald test statistic

In sensitivity analyses, excluding women who reported menstrual cycles longer than 33 days or who were parous did not change the results (data not shown). Restricting analyses to women not currently taking hormonal contraceptives at DISC06 visits did not change results substantially (data not shown). However, the positive association of premenarcheal SHBG with %DBV was attenuated and no longer statistically significant, whereas the positive association of premenarcheal DHEAS with %DBV became more linear; the geometric means of %DBV across premenarcheal DHEAS quartiles (Q1–Q4) were 16.4 %, 20.9 %, 21.7 % and 26.2 % (*P*_trend_ = 0.02). Treatment assignment did not significantly modify the association of pre- and postmenarcheal sex hormones and SHBG with %DBV (all *P*_interaction_ ≥0.11) except non-SHBG-bound estradiol (*P*_interaction_ = 0.03); however, non-SHBG-bound estradiol results were nonsignificant in either treatment group.

Because of hormonal changes with sexual maturation during adolescence [44, 45] (Table S1 in Additional file 1), we evaluated whether observed associations for DHEAS and SHBG with %DBV varied in analyses stratified by Tanner stage of breast maturation. Consistent with results by menarche status at blood collection, DHEAS and SHBG concentrations in serum collected during adolescence at Tanner breast stages 1–3 were significantly positively associated with adult %DBV, but concentrations in serum collected at breast stages 4 and 5 were not (data not shown).

Associations of sex hormones and SHBG with ADBV are shown in Tables S2 and S3 in Additional file 1 and their associations with ANDBV are shown in Table S4 and S5 in Additional file 1. Similar to %DBV, premenarcheal DHEAS and SHBG were positively associated with ADBV, but these hormones were inversely associated with ANDBV. Even so, none of the hormones or SHBG measured before menarche was significantly associated with ADBV or ANDBV. In contrast, estradiol in serum collected during adolescence after menarche in the luteal phase of the menstrual cycle was significantly positively associated with ANDBV (*P*_trend_ = 0.02); the geometric mean ANDBV increased from 294.5 to 336.0 cm^3^ between the lowest and highest estradiol quartiles.

## Discussion

In this study examining adolescent sex hormones in relation to young adult breast density, DHEAS and SHBG levels during adolescence before, but not after, menarche were significantly positively associated with adult %DBV. Estrogens, progesterone, androstenedione, and testosterone levels during adolescence were not significantly associated with adult %DBV.

It is largely unknown whether adolescent sex hormones levels predict breast cancer development later in life. Nonetheless, the breast undergoes rapid growth and development during adolescence [24, 25]. Animal and experimental studies suggest that DNA damage coupled with insufficient DNA repair during childhood or adolescence may induce epigenetic and translational modifications that have persistent effects in the breasts [48, 49]. Indeed, oral contraceptive use during teenage years [29, 50] or before pregnancy [51] was associated with higher breast tissue proliferation rates [51], higher dense breast volume [29], and elevated breast cancer risk [50].

To our knowledge, this is the first epidemiologic study that directly related adolescent sex hormone levels with adult breast density. Previous studies used sex hormone data collected from middle-aged and older women [6–21]. The association between premenarcheal DHEAS and adult %DBV in our study was nonlinearly positive. In analysis restricted to participants not using hormonal contraceptives at DISC06 visits when breast density was measured, %DBV increased monotonically across increasing quartiles of premenarcheal DHEAS from 16.4 % to 20.9 %, 21.7 %, and 26.2 %. Thus, even though we adjusted for duration of hormone use in our multivariable model, our nonlinear results might be due to residual confounding by current hormone use. This positive association is consistent with results from one study of premenopausal women [10]. Our findings for postmenarcheal DHEAS concentrations are similar to those from most studies in older women that reported relatively flat associations [12–14, 21].

The breast matures mostly before menarche under the influence of sex hormones and growth factors, and DHEAS may have its greatest effect on breast morphology at this time of expansion of the breast ductal architecture. DHEAS can be metabolized to estrogens and has been shown to have estradiol-like proliferative effects in a low-estrogen environment [52], typical of childhood and early puberty [53]. In young girls approaching puberty, prior to activation of the hypothalamic-pituitary-ovarian (HPO) axis, adrenal androgens including DHEAS can be metabolized to estrogens in adipose tissue [54]. Peripheral conversion of adrenal androgens to estrogens may explain earlier breast development independent of changes in ovarian hormones in recent birth cohorts [55] and isolated breast development that occurs without activation of the HPO in some girls [56]. DHEAS is also positively associated with growth factors during adolescence [57] that may partly regulate breast development [58–60].

Our significant positive association between premenarcheal SHBG levels and adult %DBV is consistent with some studies of premenopausal [6, 10, 11] and postmenopausal women [8, 13, 23], while others reported a suggestive, but nonsignificant positive [17, 61] or null association [14, 16, 18, 21]. Although SHBG is generally thought to modulate availability of estradiol to cells, recent discoveries of cell surface SHBG receptors and intracellular SHBG in the breast suggest that SHBG could potentially influence breast density via other mechanisms [62]. Alternatively, given that BMI is negatively correlated with breast density [63] and SHBG, our results could be due to residual confounding by BMI even though we adjusted for both childhood and adulthood BMI. This result could also be due to chance.

Notably, breast density was significantly positively associated with DHEAS and SHBG before menarche, but not after menarche. Although few prior data exist to explain our results, breast tissue typically starts to develop around 9 years of age and matures near the onset of menarche [24, 55, 64, 65]. Interestingly, in a recent large prospective cohort study, earlier thelarche (the initiation of breast development) and a longer duration between thelarche and menarche were significantly positively associated with breast cancer risk later in life [66]. The breast’s susceptibility to endogenous and possibly exogenous exposures might vary over adolescence.

Irrespective of menarche status, other sex hormones measured during adolescence were not significantly associated with adult breast density in our study, which is consistent with the largely null results reported for estrogens [8–12, 67], progesterone [8, 9, 12, 67], testosterone [10–12, 14, 16–18, 20, 22, 67, 68] and androstenedione [10, 13, 14, 18, 21] in adult women. But these hormones are primarily secreted from the ovary. Ovulatory cycles normally become stable 5 years past menarche [69], and prepubertal and early pubertal girls have relatively low ovarian hormone levels, particularly estradiol [53]. RIAs that we used to measure serum hormone levels may not be sensitive and specific enough to quantify physiologically relevant levels during early adolescence [53, 70]. The limit of detection for estradiol and estrone was 0.5 ng/dL and 43 % of girls at ≤1 year visits had estradiol levels at or below this level.

A strength of our study is the prospective design of enrolling prepubertal children, and following them to their late 20s. With repeated measurements of sex hormones, we could examine sex hormones at different stages of puberty. MRI avoids exposing participants to ionizing radiation and measures breast density not impaired by the high parenchymal breast density typical of young women [71]. Even so, breast density as measured by MRI or mammography is highly correlated (r >0.75) [72, 73], and breast density, assessed by a volumetric method or by mammography, is similarly positively associated with breast cancer risk [2, 74]. Given the natural involution of breast with aging [75], breast density measured at 25–29 years old, as in our study, might be the most relevant marker of the effect of adolescent sex hormones on the breasts, if any. Staff trained to follow a standardized protocol collected detailed information on body size, lifestyle, reproductive, and menstrual characteristics during both adolescence and adulthood, which enabled us to control for potential confounding factors.

Our study had limitations. Despite the cyclic variation of estrogens and progesterone after the onset of menarche, postmenarcheal samples were not collected timed to the menstrual cycle. Even so, luteal and follicular phase estrogens and progesterone were analyzed separately, and days until start of next menses were taken into account in analyses. Circulating sex hormone levels are an indirect marker of breast tissue exposure, but their associations with breast cancer risk indicate serum levels are a meaningful measure of exposure [76, 77]. Participants were slightly heavier with elevated LDL-C levels than the general population during childhood [33, 34], possibly limiting generalizability of our results. Sample size was relatively small. Results should be interpreted with caution because multiple tests were conducted. Finally, there is possibility of unknown residual confounding.

## Conclusions

In conclusion, circulating levels of premenarche DHEAS and SHBG were significantly positively associated with %DBV. Associations with other adolescent sex hormones were not detected. Our results suggest that differences in sex hormones during adolescence may be relevant to breast morphology during adulthood. Large studies that measure hormones using highly sensitive and specific mass spectrometry that can detect lower levels of circulating hormones in children and adolescent are warranted.

## Additional file

Additional file 1:
**Supplementary tables S1-S5.**

## Abbreviations

ADBV: absolute dense breast volume
ANDBV: absolute nondense breast volume
BMI: body mass index
CI: confidence interval
CV: coefficient of variation
%DBV: percentage dense breast volume
DHEAS: dehydroepiandrosterone sulfate
DISC: Dietary Intervention Study in Children
DISC06: Dietary Intervention Study in Children 2006 Follow-up Study
HPO: hypothalamic-pituitary-ovarian
IQR: interquartile range
LDL-C: low-density lipoprotein cholesterol
MRI: magnetic resonance imaging
QC: quality control
RIA: radioimmunoassay
SD: standard deviation
SHBG: sex hormone-binding globulin
