# Supplementary material for: Adolescent endogenous sex hormones and breast density in early adulthood
Source: Breast Cancer Res. 2015 Jun 4;17(1):77. doi: 10.1186/s13058-015-0581-4 (PMC4468804; doi:10.1186/s13058-015-0581-4)
Supplement: Additional file 1: — Supplementary tables S1-S5. [file 13058_2015_581_MOESM1_ESM.doc]

|  | **Table S1. Characteristics of study population at blood collection during adolescence ^a^** | | | | | | | | | | | | | |  |  | | | |  |
| --- | --- | --- | --- | --- | --- | --- | --- | --- | --- | --- | --- | --- | --- | --- | --- | --- | --- | --- | --- | --- |
| **Characteristics** | |  | **≤Year 1** | |  | **Year 3** | | |  | **Year 5** | | | | |  | **Last visit^b^** | | | | |
|  | |  | *N* | Mean (SD) |  | *N* | | Mean (SD) |  | *N* | Mean (SD) | | | |  | *N* | Mean (SD) | | | |
| Age at visit (yrs) | |  | 115 | 10.2 (0.6) |  | 167 | | 12.1 (0.6) |  | 143 | 14.2 (0.6) | | | |  | 143 | 16.6 (0.9) | | | |
| BMI z-score at visit | |  | 115 | 0.40 (0.89) |  | 166 | | 0.34 (0.97) |  | 143 | 0.39 (0.92) | | | |  | 142 | 0.35 (0.90) | | | |
| Days until start of next menses (days) | |  | NA |  |  | 41 | | 15 (12.6) |  | 105 | 15 (9.3) | | | |  | 119 | 16.8 (10.9) | | | |
| Physical activity (Met hours/week) | |  |  | 272.1 (47.6) |  | *159* | | 272.3 (33.8) |  | *143* | 287.7 (44.9) | | | |  | *141* | 303.4 (51.4) | | | |
| Dietary intake | |  |  |  |  |  | |  |  |  |  | | | |  |  |  | | | |
| Total calories (Kcal/day) | |  | *115* | 1584.0 (364.4) |  | *163* | | 1618.4 (450.6) |  | *137* | 1663.7 (455.5) | | | |  | *135* | 1670.1 (544.3) | | | |
| Calorie % from fat (%) | |  | *115* | 30.8 |  | *163* | | 31.2 (6.0) |  | *137* | 28.7 (6.1) | | | |  | *135* | 28.1 (7.0) | | | |
| Total dietary fiber (g/day) | |  | *115* | 6.5 (1.9) |  | *163* | | 6.5 (1.9) |  | *137* | 6.5 (1.8) | | | |  | *135* | 6.8 (2.4) | | | |
|  | |  | *N* | Percentage |  | *N* | | Percentage |  | *N* | Percentage | | | |  | *N* | Percentage | | | |
| Post- menarche at visit (%) | |  | 115 | 0% |  | 167 | | 28% |  | 143 | 85% | | | |  | 138 | 97% | | | |
| Tanner stage | |  |  |  |  |  | |  |  |  |  | | | |  |  |  | | | |
| 1 | |  | 59 | 52% |  | 14 | | 9% |  | 3 | 3% | | | |  | 0 | 0% | | | |
| 2 | |  | 48 | 42% |  | 43 | | 27% |  | 5 | 5% | | | |  | 0 | 0% | | | |
| 3 | |  | 6 | 5% |  | 50 | | 32% |  | 11 | 8% | | | |  | 0 | 0% | | | |
| 4 | |  | 1 | 1% |  | 42 | | 27% |  | 74 | 53% | | | |  | 8 | 7% | | | |
| 5 | |  | 0 | 0% |  | 9 | | 6% |  | 45 | 33% | | | |  | 109 | 93% | | | |
|  | |  |  | |  |  | | |  |  | | | | |  |  | | | | |
| **Sex hormone and SHBG** | |  | **≤Year 1** | |  | **Year 3** | | |  | **Year 5** | | | | |  | **Last visit** | | | | |
|  | |  | *N* | Median (IQR) |  | *N* | Median (IQR) | |  | *N* | Median (IQR) | | | |  | *N* | Median (IQR) | | | |
| Estradiol, pg/mL | | Premenarche | 115 | 6 (5-10) |  | 119 | 27 (9-53) | |  | 22 | 36 (26-73) | | | |  | 5 | 47 (14-52) | | | |
|  | | Follicular |  | - |  | 19 | 48 (38-51) | |  | 56 | 46 (33-83) | | | |  | 67 | 35 (24-65) | | | |
|  | | Luteal |  | - |  | 22 | 85 (57-140) | |  | 49 | 110 (51-170) | | | |  | 50 | 100 (64-160) | | | |
| Non-SHBG-bound estradiol, pg/mL | | Premenarche | 110 | 3 (2-4) |  | 119 | 14 (5-27) | |  | 22 | 20.5 (11-29) | | | |  | 5 | 15 (10-22) | | | |
|  | | Follicular |  | - |  | 19 | 25 (20-34) | |  | 56 | 25 (17-40.5) | | | |  | 67 | 21 (14-30) | | | |
|  | | Luteal |  | - |  | 22 | 44 (29-67) | |  | 49 | 53 (30-86) | | | |  | 50 | 47.5 (33-67) | | | |
| Estrone, pg/mL | | Premenarche | 115 | 14 (11-17) |  | 119 | 26 (17-36) | |  | 22 | 31.5 (19-39) | | | |  | 5 | 16 (16-30) | | | |
|  | | Follicular |  | - |  | 19 | 36 (30-41) | |  | 56 | 39 (29-56.5) | | | |  | 67 | 33 (27-42) | | | |
|  | | Luteal |  | - |  | 22 | 44 (36-51) | |  | 49 | 51 (35-80) | | | |  | 50 | 55.5 (43-81) | | | |
| Estrone sulfate, pg/mL | | Premenarche | 91 | 270 (220-350) |  | 112 | 450 (290-630) | |  | 22 | 660 (510-820) | | | |  | 5 | 440 (440-580) | | | |
|  | | Follicular |  | - |  | 18 | 725 (510-1090) | |  | 56 | 1060 (700-1575) | | | |  | 68 | 735 (580-1165) | | | |
|  | | Luteal |  | - |  | 22 | 1005 (730-1580) | |  | 49 | 1410 (1000-2690) | | | |  | 50 | 1350 (800-2380) | | | |
| Progesterone, ng/mL | | Premenarche |  | - |  |  | - | |  |  | - | | | |  | 5 | 0.2 (0.19-0.25)- | | | |
|  | | Follicular |  | - |  | 19 | 0.18 (0.11-0.22) | |  | 51 | 0.27 (0.17-0.36) | | | |  | 69 | 0.3 (0.22-0.43) | | | |
|  | | Luteal |  | - |  | 21 | 0.46 (0.27-1.43) | |  | 44 | 0.34 (0.21-3.95) | | | |  | 50 | 1.69 (0.3-5.44) | | | |
| Androstenedione, ng/dL | | Premenarche | 113 | 38 (32-58) |  | 118 | 86 (53-111) | |  | 22 | 104 (62-130) | | | |  | 5 | 126 (76-166) | | | |
|  | | Postmenarche |  | - |  | 45 | 108 (89-139) | |  | 120 | 132 (108-161.5) | | | |  | 138 | 141.5 (114-176) | | | |
| DHEAS , μg/dL | | Premenarche | 115 | 41 (26-58) |  | 120 | 57 (39.5-87) | |  | 22 | 61 (37-93) | | | |  | 5 | 143 (47-232) | | | |
|  | | Postmenarche |  | - |  | 47 | 62 (49-106) | |  | 120 | 104 (71.5-156.5) | | | |  | 138 | 150.5 (122-196) | | | |
| Testosterone, ng/dL | | Premenarche | 106 | 5.7 (3-9.2) |  | 118 | 17 (9.4-30) | |  | 22 | 24.5 (13-36) | | | |  | 5 | 37 (30-39) | | | |
|  | | Postmenarche |  | - |  | 46 | 25 (19-37) | |  | 120 | 32 (22-40) | | | |  | 136 | 33 (24.5-43) | | | |
| SHBG, nmol/L | | Premenarche | 115 | 87 (65-121) |  | 120 | 76 (45-98) | |  | 22 | 110.5 (67-146) | | | |  | 5 | 76 (70-88) | | | |
|  | | Postmenarche |  | - |  | 47 | 57 (40-83) | |  | 121 | 75 (55-99) | | | |  | 138 | 55.5 (38-74) | | | |
|  | Abbreviations: SD, standard deviation; BMI, body mass index; IQR, interquartile range; DHEAS, dehydroepiandrosterone sulfate; SHBG, sex hormone binding globulin | | | | | | | | | | | | | | | | | | | |
|  | ^a^ value for *N* for a specific year differs for different characteristics because of missing data. | | | | | | | | | | |  |  |  | | | |  |  |  |
|  | ^b^ Last visit occurred a median of 7 years after randomization in DISC | | | | | | | | | | | | |  | | | |  |  |  |

| **Table S2. Multivariate^a^ adjusted geometric mean and 95% confidence interval (CI) for absolute dense breast volume (cm^3^) by quartiles of estrogens and progesterone at pre-^b^ and postmenarcheal^c^ period** | | | | | | | | |
| --- | --- | --- | --- | --- | --- | --- | --- | --- |
|  | **Premenarche** | |  | **Postmenarche** | | | | |
| Quartiles |  |  |  | Follicular phase^d^ | |  | Luteal phase^d^ | |
|  | Mean | (95% CI) |  | Mean | (95% CI) |  | Mean | (95% CI) |
| Estradiol | | N=153 |  |  | N=110 |  |  | N=88 |
| Q1 | 80.8 | (57.6-113.3) |  | 68.6 | (46.9-100.6) |  | 73.1 | (51.1-104.7) |
| Q2 | 68.5 | (45.2-103.9) |  | 108.7 | (82.6-143.2) |  | 63.7 | (46.8-86.8) |
| Q3 | 73.9 | (56.6-96.4) |  | 67.0 | (46.8-95.8) |  | 89.8 | (62.8-128.3) |
| Q4 | 87.0 | (64.4-117.3) |  | 66.4 | (53.9-81.8) |  | 81.9 | (61.0-109.8) |
| *P-*trend^e^ | 0.50 |  |  | 0.38 |  |  | 0.09 |  |
|  |  |  |  |  |  |  |  |  |
| Non-SHBG-bound estradiol N=151 | | |  |  | N=110 |  |  | N=88 |
| Q1 | 84.5 | (57.9-123.2) |  | 72.6 | (55.9-94.2) |  | 79.7 | (58.6-108.4) |
| Q2 | 64.1 | (42.5-36.7) |  | 101.9 | (73.6-141.0) |  | 56.0 | (43.4-72.2) |
| Q3 | 75.7 | (54.5-105.1) |  | 67.7 | (58.2-78.8) |  | 93.6 | (79.3-110.6) |
| Q4 | 85.2 | (63.8-113.6) |  | 66.2 | (50.4-86.9) |  | 82.1 | (57.5-117.3) |
| *P*-trend^e^ | 0.50 |  |  | 0.30 |  |  | 0.11 |  |
|  |  |  |  |  |  |  |  |  |
| Estrone | | N=153 |  |  | N=110 |  |  | N=88 |
| Q1 | 74.8 | (48.5-115.4) |  | 66.0 | (53.6-81.2) |  | 73.3 | (63.1-85.2) |
| Q2 | 75.6 | (57.8-98.9) |  | 88.4 | (55.2-141.4) |  | 79.9 | (44.7-142.9) |
| Q3 | 77.5 | (56.5-106.3) |  | 85.7 | (68.8-106.8) |  | 79.2 | (52.7-119.0) |
| Q4 | 81.2 | (63.8-103.4) |  | 65.9 | (44.2-98.4) |  | 73.8 | (52.2-104.3) |
| *P*-trend^e^ | 0.58 |  |  | 0.88 |  |  | 0.92 |  |
|  |  |  |  |  |  |  |  |  |
| Estrone sulfate N=146 | | |  |  | N=110 |  |  | N=88 |
| Q1 | 82.8 | (55.8-123.0) |  | 77.3 | (50.0-119.6) |  | 81.2 | (49.6-133.1) |
| Q2 | 55.2 | (35.1-86.8) |  | 67.4 | (51.2-88.9) |  | 63.1 | (51.9-76.7) |
| Q3 | 80.9 | (67.0-97.6) |  | 63.8 | (48.9-83.2) |  | 92.7 | (70.7-121.7) |
| Q4 | 91.6 | (73.4-114.3) |  | 96.7 | (72.4-129.1) |  | 72.1 | (56.4-92.1) |
| *P*-trend^e^ | 0.27 |  |  | 0.61 |  |  | 0.95 |  |
|  |  |  |  |  |  |  |  |  |
| Progesterone | |  |  |  | N=110 |  |  | N=84 |
| Q1 | – |  |  | 66.5 | (47.4-93.4) |  | 74.8 | (59.3-94.5) |
| Q2 | – |  |  | 89.9 | (69.3-116.6) |  | 72.6 | (53.9-97.9) |
| Q3 | – |  |  | 78.0 | (57.3-106.2) |  | 79.3 | (48.2-130.5) |
| Q4 | – |  |  | 68.2 | (51.2-90.8) |  | 81.8 | (49.5-135.2) |
| *P*-trend^e^ |  |  |  | 0.99 |  |  | 0.32 |  |
| Abbreviations: SHBG, sex hormone binding globulin | | | | | | | |  |
| ^a^ Geometric means and 95% CI are estimated from linear mixed effects models including clinic as a random effect and adjusted for treatment group (diet intervention group, usual-care control group), BMI (kg/m^2^, continuous), parity (0 and >0), duration of hormone use (yrs, continuous), race (White and non-White), and education (bachelor's degree, graduate school and other) as fixed effects | | | | | | | | |
| ^b^ The premenacheal estrogen levels were standardized by adjusting for age at visit, BMI Z-score at visit, and visit number in years after randomization by residual method. | | | | | | | | |
| ^c^ The postmenacheal estrogens and progesterone levels were standardized by adjusting for age at visit, BMI Z-score at visit, visit number in years after randomization and days from blood draw to start of next menses as a cubic spline by residual method. | | | | | | | | |
| ^d^. Days 1 through 14 before next menses were defined as luteal, whereas day 0 and days greater than 14 were defined as follicular. | | | | | | | | |
| ^e^ *P*-test for trend was conducted by modeling the quartile medians of standardized hormone concentrations as a continuous term in linear mixed effects models and calculating the Wald test statistic. | | | | | | | | |

| **Table S3. Multivariate^a^ adjusted geometric mean and 95% confidence interval (CI) for absolute dense breast volume (cm^3^) by quartiles of androgens and SHBG at pre-^b^ and postmenarcheal^b^ period** | | | | | | | | |
| --- | --- | --- | --- | --- | --- | --- | --- | --- |
| Quartiles | **Premenarche** | |  | **Postmenarche** | |  | **All periods** | |
|  | Mean | (95% CI) |  | Mean | (95% CI) |  | Mean | (95% CI) |
| Androstenedione N=153 | | |  |  | N=154 |  |  | N=174 |
| Q1 | 86.5 | (72.4-103.4) |  | 75.7 | (66.0-86.8) |  | 85.3 | (72.3-100.5) |
| Q2 | 79.5 | (63.1-100.2) |  | 80.2 | (65.1-98.8) |  | 79.0 | (57.5-108.6) |
| Q3 | 75.1 | (49.1-115.0) |  | 78.0 | (59.2-102.6) |  | 75.7 | (64.1-89.3) |
| Q4 | 68.7 | (46.3-101.9) |  | 72.3 | (51.0-102.6) |  | 74.4 | (56.7-97.5) |
| *P*-trend^c^ | 0.13 |  |  | 0.77 |  |  | 0.30 |  |
|  |  |  |  |  |  |  |  |  |
| DHEAS | | N=153 |  |  | N=156 |  |  | N=176 |
| Q1 | 72.4 | (49.2-106.6) |  | 73.8 | (50.4-108.2) |  | 76.8 | (58.0-101.5) |
| Q2 | 81.1 | (56.4-116.8) |  | 73.2 | (50.4-106.4) |  | 74.9 | (59.9-93.7) |
| Q3 | 69.9 | (51.7-94.5) |  | 74.6 | (65.3-85.1) |  | 76.6 | (64.5-91.1) |
| Q4 | 86.8 | (75.6-99.6) |  | 86.8 | (76.5-98.4) |  | 87.6 | (78.3-98.1) |
| *P*-trend^c^ | 0.19 |  |  | 0.47 |  |  | 0.31 |  |
|  |  |  |  |  |  |  |  |  |
| Testosterone N=150 | | |  |  | N=156 |  |  | N=175 |
| Q1 | 74.1 | (58.7-93.4) |  | 70.1 | (60.2-81.6) |  | 78.8 | (61.2-101.3) |
| Q2 | 89.4 | (79.6-100.5) |  | 83.4 | (66.5-104.7) |  | 85.9 | (73.4-100.6) |
| Q3 | 73.7 | (44.0-123.5) |  | 78.7 | (54.5-113.5) |  | 78.3 | (60.3-101.5) |
| Q4 | 72.0 | (53.0-97.8) |  | 76.0 | (58.9-98.1) |  | 72.6 | (55.1-95.6) |
| *P*-trend^c^ | 0.76 |  |  | 0.71 |  |  | 0.57 |  |
|  |  |  |  |  |  |  |  |  |
| SHBG | | N=153 |  |  | N=156 |  |  | N=176 |
| Q1 | 61.1 | (42.8-87.4) |  | 66.3 | (44.8-98.4) |  | 62.7 | (51.4-76.5) |
| Q2 | 73.8 | (54.3-100.2) |  | 72.8 | (55.3-95.7) |  | 78.9 | (59.1-105.4) |
| Q3 | 82.0 | (67.4-99.8) |  | 105.2 | (86.3-128.2) |  | 93.1 | (85.0-101.9) |
| Q4 | 96.8 | (76.2-122.9) |  | 68.9 | (55.7-85.3) |  | 83.8 | (65.2-107.7) |
| *P*-trend^c^ | 0.08 |  |  | 0.60 |  |  | 0.08 |  |
| Abbreviations: DHEAS, dehydroepiandrosterone sulfate; SHBG, sex hormone binding globulin | | | | | | | | |
| ^a^ Geometric means and 95% CI are estimated from linear mixed effects models including clinic as a random effect and adjusted for treatment group (diet intervention group, usual-care control group),, BMI (kg/m^2^, continuous), parity (0 and >0), duration of hormone use (yrs, continuous), race (White and non-White), and education (bachelor's degree, graduate school and other) as fixed effects | | | | | | | | |
| ^b^ The androgens and SHBG levels were standardized by adjusting for age at visit, BMI Z-score at visit, and visit number in years after randomization by residual method. | | | | | | | | |
| ^c^ *P*-test for trend was conducted by modeling the quartile medians of standardized hormone concentrations as a continuous term in linear mixed effects models and calculating the Wald test statistic. | | | | | | | | |

| **Table S4. Multivariate^a^ adjusted geometric mean and 95% confidence interval (CI) for absolute nondense breast volume (cm^3^) by quartiles of estrogens and progesterone at pre-^b^ and postmenarcheal^c^ period** | | | | | | | | |
| --- | --- | --- | --- | --- | --- | --- | --- | --- |
|  | **Premenarche** | |  | **Postmenarche** | | | | |
| Quartiles |  |  |  | Follicular phase^d^ | |  | Luteal phase^d^ | |
|  | Mean | (95% CI) |  | Mean | (95% CI) |  | Mean | (95% CI) |
| Estradiol | | N=153 |  |  | N=110 |  |  | N=88 |
| Q1 | 295.8 | (275.7-317.3) |  | 286.3 | (236.3-346.8) |  | 294.5 | (286.5-302.8) |
| Q2 | 251.8 | (230.9-274.7) |  | 288.6 | (236.6-352.0) |  | 317.5 | (255.8-394.2) |
| Q3 | 290.8 | (231.8-364.8) |  | 302.6 | (252.4-362.9) |  | 320.8 | (265.5-387.6) |
| Q4 | 313.3 | (261.9-374.8) |  | 267.9 | (198.7-361.2) |  | 336.0 | (289.3-390.3) |
| *P-*trend^e^ | 0.40 |  |  | 0.75 |  |  | 0.02 |  |
|  |  |  |  |  |  |  |  |  |
| Non-SHBG-bound estradiol N=151 | | |  |  | N=110 |  |  | N=88 |
| Q1 | 299.9 | (275.0-327.1) |  | 291.2 | (228.4-371.2) |  | 292.6 | (267.7-319.9) |
| Q2 | 249.2 | 220.9-281.1) |  | 280.9 | (261.9-301.2) |  | 347.0 | (309.5-388.9) |
| Q3 | 282.2 | (281.4-364.6) |  | 315.3 | (299.1-332.4) |  | 294.3 | (231.0-375.0) |
| Q4 | 326.4 | (257.8-413.3) |  | 259.2 | (197.1-340.7) |  | 337.3 | (291.1-391.0) |
| *P*-trend^e^ | 0.33 |  |  | 0.67 |  |  | 0.48 |  |
|  |  |  |  |  |  |  |  |  |
| Estrone | | N=153 |  |  | N=110 |  |  | N=88 |
| Q1 | 290.9 | (255.5-331.1) |  | 312.0 | (254.0-383.3) |  | 306.2 | (252.7-371.0) |
| Q2 | 274.6 | (249.3-302.5) |  | 280.0 | (220.1-356.2) |  | 319.8 | (262.2-390.0) |
| Q3 | 279.5 | (236.3-330.6) |  | 304.8 | (271.7-341.9) |  | 333.3 | (268.8-413.4) |
| Q4 | 304.1 | (248.3-372.4) |  | 250.7 | (174.9-359.3) |  | 308.9 | (268.0-355.9) |
| *P*-trend^e^ | 0.65 |  |  | 0.43 |  |  | 0.83 |  |
|  |  |  |  |  |  |  |  |  |
| Estrone sulfate N=146 | | |  |  | N=110 |  |  | N=88 |
| Q1 | 284.3 | (263.6-306.7) |  | 260.3 | (206.1-328.8) |  | 276.4 | (244.0-313.0) |
| Q2 | 298.9 | (227.7-392.3) |  | 311.3 | (266.8-363.2) |  | 346.2 | (282.1-424.9) |
| Q3 | 279.9 | (233.0-336.2) |  | 288.3 | (235.2-353.3) |  | 360.4 | (297.1-437.2) |
| Q4 | 282.8 | (221.2-361.7) |  | 280.7 | (214.9-366.9) |  | 292.3 | (230.5-370.7) |
| *P*-trend^e^ | 0.86 |  |  | 0.76 |  |  | 0.41 |  |
|  |  |  |  |  |  |  |  |  |
| Progesterone | |  |  |  | N=110 |  |  | N=84 |
| Q1 | – |  |  | 292.3 | (224.3-380.9) |  | 340.2 | (305.2-379.2) |
| Q2 | – |  |  | 281.8 | (239.9-331.0) |  | 306.7 | (251.5-374.2) |
| Q3 | – |  |  | 276.2 | (215.7-353.6) |  | 266.6 | (218.4-325.5) |
| Q4 | – |  |  | 318.5 | (242.9-417.6) |  | 341.1 | (268.6-433.1) |
| *P*-trend^e^ |  |  |  | 0.61 |  |  | 0.68 |  |
| Abbreviations: SHBG, sex hormone binding globulin | | | | | | | |  |
| ^a^ Geometric means and 95% CI are estimated from linear mixed effects models including clinic as a random effect and adjusted for treatment group (diet intervention group, usual-care control group), BMI (kg/m^2^, continuous), parity (0 and >0), duration of hormone use (yrs, continuous), race (White and non-White), and education (bachelor's degree, graduate school and other) as fixed effects | | | | | | | | |
| ^b^ The premenacheal estrogen levels were standardized by adjusting for age at visit, BMI Z-score at visit, and visit number in years after randomization by residual method. | | | | | | | | |
| ^c^ The postmenacheal estrogens and progesterone levels were standardized by adjusting for age at visit, BMI Z-score at visit, visit number in years after randomization and days from blood draw to start of next menses as a cubic spline by residual method. | | | | | | | | |
| ^d^. Days 1 through 14 before next menses were defined as luteal, whereas day 0 and days greater than 14 were defined as follicular. | | | | | | | | |
| ^e^ *P*-test for trend was conducted by modeling the quartile medians of standardized hormone concentrations as a continuous term in linear mixed effects models and calculating the Wald test statistic. | | | | | | | | |

| **Table S5. Multivariate^a^ adjusted geometric mean and 95% confidence interval (CI) for absolute nondense breast volume (cm^3^) by quartiles of androgens and SHBG at pre-^b^ and postmenarcheal^b^ period** | | | | | | | | |
| --- | --- | --- | --- | --- | --- | --- | --- | --- |
| Quartiles | **Premenarche** | |  | **Postmenarche** | |  | **All periods** | |
|  | Mean | (95% CI) |  | Mean | (95% CI) |  | Mean | (95% CI) |
| Androstenedione N=153 | | |  |  | N=154 |  |  | N=174 |
| Q1 | 293.7 | (274.2-314.5) |  | 297.3 | (245.8-359.6) |  | 306.2 | (279.4-335.4) |
| Q2 | 279.7 | (234.4-333.8) |  | 271.1 | (228.4-321.6) |  | 270.8 | (245.9-298.1) |
| Q3 | 289.0 | (252.0-331.3) |  | 310.2 | (251.4-382.8) |  | 299.3 | (234.7-381.6) |
| Q4 | 285.9 | (215.0-380.4) |  | 268.2 | (220.2-326.8) |  | 269.5 | (224.3-323.9) |
| *P*-trend^c^ | 0.88 |  |  | 0.71 |  |  | 0.44 |  |
|  |  |  |  |  |  |  |  |  |
| DHEAS | | N=153 |  |  | N=156 |  |  | N=176 |
| Q1 | 294.3 | (257.3-336.6) |  | 248.4 | (221.0-279.1) |  | 271.9 | (247.8-298.4) |
| Q2 | 277.0 | (226.6-338.5) |  | 298.3 | (261.8-339.8) |  | 287.8 | (249.8-331.6) |
| Q3 | 312.7 | (276.1-354.2) |  | 318.9 | (259.1-392.5) |  | 322.8 | (271.2-384.1) |
| Q4 | 266.3 | (224.0-316.4) |  | 293.6 | (254.1-339.1) |  | 272.7 | (244.2-305.6) |
| *P*-trend^c^ | 0.39 |  |  | 0.06 |  |  | 0.51 |  |
|  |  |  |  |  |  |  |  |  |
| Testosterone N=150 | | |  |  | N=156 |  |  | N=175 |
| Q1 | 288.3 | (257.4-322.8) |  | 307.8 | (262.6-360.7) |  | 292.3 | (250.2-341.5) |
| Q2 | 296.8 | (251.9-349.6) |  | 310.9 | (289.2-334.1) |  | 313.7 | (278.9-352.8) |
| Q3 | 283.4 | (236.2-340.2) |  | 288.7 | (231.2-360.5) |  | 276.4 | (214.7-355.9) |
| Q4 | 288.7 | (216.7-384.6) |  | 251.1 | (217.0-290.5) |  | 272.7 | (234.5-317.2) |
| *P*-trend^c^ | 0.96 |  |  | 0.08 |  |  | 0.55 |  |
|  |  |  |  |  |  |  |  |  |
| SHBG | | N=153 |  |  | N=156 |  |  | N=176 |
| Q1 | 306.8 | (271.2-347.1) |  | 294.1 | (258.5-334.7) |  | 283. 8 | (250.6-321.5) |
| Q2 | 270.7 | (230.2-318.2) |  | 308.6 | (258.8-367.9) |  | 326.6 | (279.0-382.2) |
| Q3 | 306.8 | (245.7-383.0) |  | 251.0 | (198.1-318.1) |  | 256.5 | (208.6-315.5) |
| Q4 | 266.1 | (234.5-301.9) |  | 304.4 | (248.0-373.6) |  | 289.7 | (248.4-337.9) |
| *P*-trend^c^ | 0.32 |  |  | 0.84 |  |  | 0.62 |  |
| Abbreviations: DHEAS, dehydroepiandrosterone sulfate; SHBG, sex hormone binding globulin | | | | | | | |  |
| ^a^Geometric means and 95% CI are estimated from linear mixed effects models including clinic as a random effect and adjusted for treatment group (diet intervention group, usual-care control group), BMI (kg/m^2^, continuous), parity (0 and >0), duration of hormone use (yrs, continuous), race (White and non-White), and education (bachelor's degree, graduate school and other) as fixed effects | | | | | | | | |
| ^b^ The androgens and SHBG levels were standardized by adjusting for age at visit, BMI Z-score at visit, and visit number in years after randomization by residual method. | | | | | | | | |
| ^c^ *P*-test for trend was conducted by modeling the quartile medians of standardized hormone concentrations as a continuous term in linear mixed effects models and calculating the Wald test statistic. | | | | | | | | |
